# Prescribing Habits of Clinicians and Medication Journey of Patients Treated for Attention Deficit Hyperactivity Disorder (ADHD): Experience From a Large London Clinic

**DOI:** 10.1192/bjo.2024.697

**Published:** 2024-08-01

**Authors:** Azizah Attard, Jessie Pang, Stephen Attard, Hugo de Waal

**Affiliations:** 1 Berkeley Psychiatrists, London, United Kingdom; 2 West London NHS Trust, London, United Kingdom; 3 CNWL, London, United Kingdom

## Abstract

**Aims:**

To understand the prescribing habits and trends of clinicians in a large ADHD clinic and the medication journey of patients from point of diagnosis to the point of agreeing a shared care plan with primary care services.

**Methods:**

This was a non-interventional retrospective study collecting information from anonymised electronic patient and prescription records. Following approval by the Clinical Governance body of the practice, in June 2023, all patients with a SCP between the years 2019 and 2021 were identified. Data collected included patient demographics, date that medication was started, discontinued, or switched along with associated reasons. Additionally, to better understand the time taken to gain publication of a SCP, the amount of clinician-patient facing time was recorded, including the number of brief follow-up appointments, number of repeat prescriptions and number of clinician to patient emails. Patient data was fully anonymised and any identifiable data removed.

**Results:**

All but one patient was started on a stimulant medication immediately following diagnosis, in line with national prescribing guidance. Atomoxetine was the medication of choice for a patient with a previous history of stimulant intolerance. 74% (n = 95) of patients were started on lisdexamfetamine, 20% (n = 25) were started on methylphenidate long-acting formulations and five patients on short-acting methylphenidate agent.

Of the 95 patients initiated on lisdexamfetamine, 78 (82.1%) were continued on lisdexamfetamine until the point of publication of their Shared Care Policy (SCP), nine (9.5%) patients switched medication, 10 patients initiated additional fast-acting dexamfetamine tablets and two had a second agent added to their therapy (atomoxetine n = 1, methylphenidate n = 1).

Of 25 patients initiated on methylphenidate long acting, 18 (72%) continued this medication at the point of publication of SCP. Of 25 patients initiated on methylphenidate long acting, seven (28%) patients switched medication and two patients were initiated on an additional fast acting methylphenidate.

To successfully stabilise and publish 134 patients on a SCP, 404 brief follow up appointments of 15-minute duration were utilised, which totals 6060 minutes of patient facing time. Of 134 patients, most n = 41 (30.6%) had 2 brief follow-up appointments; 32 (23.9%) had 3 brief follow up appointments and 22 (16.4%) had 4 appointments. Two patients did not attend a follow up appointment, and one patient had 11 brief follow up appointments.

**Conclusion:**

Stimulant medications were typically used as first line treatment however, of these 74% were started on lisdexamfetamine while only 20% were initiated on long acting methylphenidate. Those started on lisdexamfetamine were more likely to continue on this medication to the point shared care agreement than those started on methylphenidate, 28% of whom switched to an alternative, for a variety of reasons. Mean time to reading a shared care agreement was longer for those initiated on methylphenidate long acting compared to lisdexamfetamine.

The data show that for most patients the journey from initiation of a stimulant medication to a shared care agreement was a straightforward one, with the majority having either two or three follow up appointments.

More research is needed to better understand the apparent differences in pathway for those commenced on lisdexamfetamine and long acting methylphenidate.

